# A gaping gap (smokeless tobacco control in Pakistan)

**DOI:** 10.1186/s12971-016-0102-y

**Published:** 2016-11-21

**Authors:** Zohaib Khan

**Affiliations:** 1 Leibniz Institute for Prevention Research and Epidemiology-BIPS GmbH, Achterstrasse 30, 28359 Bremen, Germany; 2 Khyber Medical University, Peshawar, Pakistan

**Keywords:** Smokeless tobacco, Youth, Oral cancer, Tobacco control, FCTC, Sales ban

## Abstract

Oral cancer is second most common cancer in Pakistan and one of the major contributing factors to its high incidence is smokeless tobacco (SLT) use. 5.3% of Pakistan’s youth are current SLT users. The World Health Organization requires the signatories of its “Framework Convention on Tobacco Control” to officially ban the sale of tobacco products to minors. We reviewed the Government of Pakistan’s tobacco control, and related supporting policies, to assess how these address the issue of sale of SLT products to minors and found evident gaps in this regard. Legislations need to be in place to ban the sale of SLT products to minors and avoid an SLT epidemic in the future.

## Background

Smokeless tobacco is associated with a variety of oral and systemic disease [1, 2], in particular with oral and pharyngeal cancers [3]. Pakistan has one of the highest incidence rates of oral cancer in the world [4]. It is the most common cancer among men and the second most common cancer among women in the country [5]. Tobacco use and alcohol consumption are considered as the leading modifiable risk factors for oral cancer and account for over 70% of the population attributable fraction for oral cancer [6]. A World Health Organization (WHO) report from 2001 suggests that Pakistan has one of the lowest per capita consumption of alcohol in the world [7], which might be due to a public ban on consumption and sale of alcohol. Tobacco, therefore, seems to be a major reason for the high incidence of oral cancer in Pakistan, this is substantiated by recent evidence from systematic reviews of literature pertaining to South Asia which implies that SLT is one of the main factors responsible for a high incidence of oral cancer in the region [8, 9]. Results from the Global Youth Tobacco Survey (GYTS), carried out recently in Pakistan, show that 5.3% (approx. 4.2 million) of the country’s youth currently use smokeless tobacco (SLT) products and another 4.7% were past users [10]. The results from GYTS are particularly alarming because the survey sample consisted of school going children aged 13–15 years. Evidence shows that a social disparity exist with regards to both oral cancer incidence and SLT use i.e., People from low socioeconomic status, and lower or no education level, are at a higher of oral cancer and having an SLT habit [11]. This could imply, that potentially the actual prevalence of SLT use might even be higher among the youth of Pakistan, given that the national literacy rate is just 46% [12].

Pakistan is a signatory of the WHO’s Framework Convention for Tobacco Control (FCTC) since 2005, and has taken significant steps to curb smoking in the country [13]. Article 16 of the FCTC states, “Each Party shall adopt and implement effective legislative, executive, administrative or other measures at the appropriate government level to prohibit the sales of tobacco products to persons under the age set by domestic law, national law or eighteen”, an intervention aimed at curbing the use of tobacco among minors. However, the results of the GYTS and evidence from more recent studies [14, 15], suggest that tobacco control in Pakistan may be lagging in its effectiveness to reduce the prevalence of tobacco products use among minors.

A 2014 research article explored public policy gaps with regards to SLT control in four Asian countries including Pakistan, by conducting a review of policy documents and interviews with key informants [16], one of the findings of this study was that, “the sale of smokeless tobacco to and by minors is prohibited in Pakistan”. The dichotomy between the alarming prevalence of SLT use among minors in Pakistan and the reported findings of the aforementioned policy review study [16], warranted a review of the Government of Pakistan policies to identify, how the issue of sale to and by, and consumption of SLT by minors has been addressed in these policy documents.

## Methods

We downloaded the official documents related to tobacco control from the official website of the Government of Pakistan’s Tobacco Control Cell, a body responsible for research, advocacy and legislation pertaining to tobacco control in Pakistan. Documents pertaining to child labor in Pakistan were downloaded from the United Nations’ International labor Organization’s “database of national labor, social security and related human rights legislation (NATLEX)” database. Provincial policy documents, if applicable, were downloaded from the official websites of the respective provinces of Pakistan. In order to supplement the information from the official documents, we performed an electronic search in Medline via PubMed using various combinations of MeSH terms and keywords, to identify tobacco control policy related literature from Pakistan. The final search query used in PubMed was ((“pakistan”[MeSH Terms] OR “pakistan”[All Fields]) AND (“tobacco”[MeSH Terms] OR “tobacco”[All Fields] OR “tobacco products”[MeSH Terms] OR (“tobacco”[All Fields] AND “products”[All Fields]) OR “tobacco products”[All Fields])) AND (“policy”[MeSH Terms] OR “policy”[All Fields]). From the retrieved articles those which reviewed, or were specifically aimed at informing public tobacco control policy in Pakistan, were included in this review. Additionally, an email request was sent to the authors of the 2014, SLT control policy review article, to explain the basis of their findings regarding the prohibition of sale of SLT to and by minors in Pakistan.

## Results

Table 1 refers to key findings from the relative official documents of the Government of Pakistan. The PubMed search returned 27 articles out of which two were eligible to be included in this review. The first article was the afore-cited review of the SLT control policies in South Asia which had concluded that SLT sale to and by minors is prohibited in Pakistan [16]. The second article was based on a core Non-communicable disease prevention policy document from Pakistan and only addressed the prohibition of sale and consumption of cigarettes to minors, without any mention of SLT use among minors [17]. In response to the authors’ query, the authors of the article on SLT control policies in South Asia [16] cited “section 9” of the “Prohibition of Smoking and Protection of Non-Smokers Health Ordinance 2002”, and information from a key personnel that the child labor act prohibits children from working (Including selling tobacco products), as the basis of their finding, that sale of SLT products to and by minors in Pakistan is prohibited.

**Table 1:** Smokeless tobacco control and minors in Pakistan

No	Document name	Available at	Relevant sections/articles/paragraphs	“SLT and minors” related findings
	Prohibition of Smoking and Protection of Non-Smokers Health Ordinance 2002.	http://www.tcc.gov.pk/downloads.php	• Section 8, titled *Prohibition of sale of cigarettes, etc. to minors-* “No person shall sell cigarettes or any other smoking substance to any person who is below the age of eighteen years”. • Section 9, “*Prohibition of storage, sale and distribution of cigarettes, etc.,in the immediate vicinity of educational institutions”* – No person shall himself or by any person on his behalf, store, sell or distribute cigarettes or any other smoking substance or any other tobacco product within (fifty) meters from any college, school or educational institution. • Explanation of Educational institutions: “All schools in the country, Primary, Secondary, Higher Secondary, all Colleges, Intermediate degree, Medical Colleges, Engineering Colleges, Agriculture and all other institutions and all the Universities, private or Government have declared 100% smoke free. No student, teacher, persons who are working in the above mentioned institutions and all heads of institutions cannot smoke in the premises”. **“**No tobacco products can be sold within the 50 meters area of the premises of all above mentioned”	• Sale of cigarettes and other smoking substances to minors is banned, no mention of SLT. • Selective prohibition i.e., Ban on sale of all kinds of tobacco in and in the immediate vicinity of various institutions. • All institutions mentioned are declared “smoke free” and not “tobacco free”.
	SRO on “The Prohibition of Sale of Cigarettes to Minors Rules, 2010	http://www.tcc.gov.pk/downloads.php	• No mention of smokeless tobacco.	• None
	Monitoring Checklist for Implementation Committee, 2002	http://www.tcc.gov.pk/downloads.php	• Section 8, Sale of cigarettes and other tobacco products to minors Yes/No • Section 9, Presence or absence of cigarette sales outlet/s within 50 m of the institution, and the presence or absence of cigarette sale in the institutions cantine.	• Monitoring of tobacco products sale to minors. • Monitoring of sale of tobacco products in, or in the vicinity, of institutions mentioned in section 9 of the 2002 ordinance.
	Media Resource Kit on Tobacco Control	http://www.tcc.gov.pk/downloads.php	The media tool kit has a section on SLT, however, there are no particular references to minors.	• None.
	Employment of children act, 1991^a^	http://www.na.gov.pk/uploads/documents/1335242011_887.pdf	• Section 3. *Prohibition of Employment.* “No child shall be employed or permitted to work in any of the occupations set forth in Part I of the Schedule or in any workshop wherein any of the processes set forth in Part II of that Schedule is carried on: Tobacco processing and manufacturing including niswar^c^ and bidi^b^ making.” Is mentioned as one of the processes (part II) where child employment is illegal.	• None • Children under 14 years of age are not allowed to be involved in processing or manufacturing of tobacco.
	The Khyber Pakhtunkhwa Prohibition of Employment of Children Act, 2015 (Act No. XIX of 2015).	http://www.pakp.gov.pk/2013/acts/the-khyber-pakhtunkhwa-prohibition-of-employment-of-children-act-2015/	• *Section 3. Prohibition of employment.* “(1) No child shall be employed or permitted to work in any establishment: Provided that a child not below the age of 12 years may be engaged in the light work, alongside his family member, for a maximum of two hours per day mainly for the purpose of acquiring skills, in a private undertaking, or in any school established, assisted or recognized by Government for such purpose. (2) No adolescent shall be employed or permitted to work in any hazardous work included in the Schedule”. The above mentioned “Schedule” includes “Tobacco processing and manufacturing including Niswar and bidi making”.	• No children or adolescent is allowed to work in tobacco processing and manufacturing.
	Shops and Establishments Ordinance 1969	http://www.ilo.org/dyn/travail/docs/1008/West%20Pakistan%20Shops%20and%20Establishments%20Ordinance%201969.pdf	*Section 20. Prohibition of employment of children*. “No child shall be required or allowed to work in any establishment” *Section 7. Opening and closing hours of establishments* “Except with the permission of Government, no woman or young person shall be employed in any establishment otherwise than between the hour of 9–00 a.m. to 7:00 p.m”.	Children under 14 years are not allowed to work in any establishment, which may also include tobacco sale shops.
	Government of Sindh Resolution, PAS/Legis-R-202/2016/734-A	http://www.pas.gov.pk/index.php/mediacenter/ntf/en/31/1404	Resolution- “This Assembly resolves that Provincial Government impose ban on import, sale and purchase of beetal nut & Gutka in the entire Province of Sindh. According to medical statistics chewing Gutka is injurious to health and is pre-cancerous”.	• Selective ban on SLT i.e., Gutka^c^ only.

^a^Amended version (via SRO 387)

^b^Hand rolled cigarettes comprising of tobacco wrapped in a plant leaf

^c^Different forms of SLT

## Discussion

The findings from table 1 clearly suggest gaps in public policy with regards to SLT sale, to and by minors. The “section 8” of the 2002 Ordinance only focuses on “smoking tobacco” products sales to and by minors without mentioning smokeless tobacco. On the contrary, the monitoring tool designed to assess the implementation of the same ordinance assesses the sale of both smoking and other forms of tobacco, to and by minors, which implies a disconnect between the two documents. The 2002 ordinance also puts a selective ban on the sale of any tobacco products to any age group i.e., only inside and in the near vicinity (50 m) of educational and public sector institutions, and renders these public and educational buildings “smoke free”. This would technically imply that a person, irrespective of age, can consume smokeless tobacco within these institutions. Additionally, given that 54% of Pakistan’s population is not literate [12], the potential applicability of the section 9 of the ordinance may only be limited to half of the country’s population. In Sindh province there is a selective ban on manufacture and sale of Gutka but other forms of SLT are still manufactured and sold. In Khyber Pakhtunkhwa both children and adolescents are not allowed to work in the manufacture or processing of Naswar but there are no provisions for prohibition of sale of Naswar to and by minors.

With regards to the difference between our finding and those reported by Khan et al. [16]. We believe that the basis on which they concluded that “In Pakistan, the sale of SLT to and by minors is prohibited”, are too vague and open to interpretation, to draw a solid conclusion from. Firstly, “section 9” of the 2002 ordinance puts only a “geographically limited” ban/prohibition on the sale of tobacco products in, or in the near vicinity of selected public/private buildings. Given that section 8 of the ordinance only prohibits sale of cigarettes and other smoking substances (not SLT) to and by minors, thus, technically a minor could sell or buy an SLT product at a 51 m distance from the institutions/buildings mentioned in the ordinance. Secondly, the national act related to child labor in Pakistan explicitly bans children from selected “occupations and processes”, none of which are related to SLT sale. It is also pertinent to note that apart from the Khyber Pakhtunkhwa province, where adolescents are also covered by its own child labor law, the national act is applicable only to children under the age of 14, while “minor” has been defined as a person under the age of 18 in the official documents of the Tobacco Control Cell of Pakistan. The “shops and establishments act” is also aimed at children under the age of 14, prohibiting them to work in any establishment. Again the word “establishment” is not clearly defined in the ordinance and may or may not be applicable to shops where tobacco is sold.

From these findings it is evident that there are gaps regarding children and particularly “adolescents”, in the SLT control policies of Pakistan. Adolescence is considered one of the most vulnerable age groups for tobacco uptake and therefore shall be one of the primary targets of a tobacco control policies and interventions [18]. From the review of the official documents of the Government of Pakistan, we can also infer that most of the focus is on the supply side i.e., manufacture and sale of tobacco products, with no legislations regarding the demand side i.e., Possession, use, and purchase (PUP) of tobacco products by minors. Sufficient evidence exists that PUP laws aimed at reducing access to tobacco products are an effective adjuvant to any tobacco control measures [19].

### Conclusions and policy implications

From the results and discussion we can conclude that a differential focus with regards to smoking and smokeless tobacco products exists in the current tobacco control policies in Pakistan. There is also some evidence that the tobacco industry as well as some part of the scientific community suggest SLT use as means of harm reduction [20], which might lead to an increase in the uptake of SLT products in Pakistan. As such, this warrants a non-differential focus on both smoking as well as SLT products from the Government of Pakistan and calls for fashioning of legislative measures, aimed to curb both the sale and consumption of SLT products among minors. Some of the gaps identified above are technical and others need more explicit explanation or clarification. The sale and consumption of SLT by minors has not been adequately and explicitly addressed in the relevant legislation. The supporting laws in tobacco control e.g., “Employment of children act”, may need revisions and must also address adolescents.

## Abbreviations

FCTC: Framework Convention on Tobacco Control
GYTS: Global Youth Tobacco Survey
SLT: Smokeless tobacco
SRO: Statutory rules and orders
TCC: Tobacco Control Cell (Ministry of National Health Services, Regulations and Coordination Government of Pakistan)
WHO: World Health Organization

## References

[1] Critchley JA, Unal B (2003). Health effects associated with smokeless tobacco: a systematic review. Thorax.

[2] Lee PN, Hamling J (2009). Systematic review of the relation between smokeless tobacco and cancer in Europe and North America. BMC Med.

[3] Boffetta P, Hecht S, Gray N, Gupta P, Straif K (2008). Smokeless tobacco and cancer. Lancet Oncol.

[4] de Camargo CM, Voti L, Guerra-Yi M, Chapuis F, Mazuir M, Curado MP (2010). Oral cavity cancer in developed and in developing countries: population-based incidence. Head Neck.

[5] Ferlay J, Soerjomataram I, Ervik M, Dikshit R, Eser S, Mathers C, Rebelo M, Parkin DM, Forman D, Bray, F. GLOBOCAN 2012 v1.0, Cancer Incidence and Mortality Worldwide: IARC CancerBase No. 11 [Internet]. Lyon, France: International Agency for Research on Cancer; 2013. Available from: http://globocan.iarc.fr. Accessed 18 Nov 2016.

[6] Radoi L, Paget-Bailly S, Cyr D, Papadopoulos A, Guida F, Schmaus A, Cenee S, Menvielle G, Carton M, Lapotre-Ledoux B, Delafosse P, Stuecker I, Luce D (2013). Tobacco smoking, alcohol drinking and risk of oral cavity cancer by subsite: results of a French population-based case-control study, the ICARE study. Eur J Cancer Prev.

[7] World Health Organization (2001). Alcohol per capita consumption, patterns of drinking and abstention worldwide after 1995. appendix 2. Eur Addict Res.

[8] Khan Z, Tonnies J, Muller S (2014). Smokeless tobacco and oral cancer in South Asia: a systematic review with meta-analysis. J Cancer Epidemiol.

[9] Guha N, Warnakulasuriya S, Vlaanderen J, Straif K (2014). Betel quid chewing and the risk of oral and oropharyngeal cancers: a meta‐analysis with implications for cancer control. Int J Cancer.

[10] Fact sheets Pakistan—Karachi C. Global Youth Tobacco Survey (GYTS). Geneva: World Health Organization. 2013; Available at http://www.emro.who.int/images/stories/tfi/documents/GYTS_FS_PAK_2013.pdf?ua=1. Accessed on 29 Aug 2016.

[11] Conway DI, Petticrew M, Marlborough H, Berthiller J, Hashibe M, Macpherson LM (2008). Socioeconomic inequalities and oral cancer risk: a systematic review and meta-analysis of case-control studies. Int J Cancer.

[12] Latif A. Alarming situation of education in Pakistan. Press International Report. 2011. Available at http://www.closer2you.net/Education_EN.pdf. Accessed 18 Nov 2016.

[13] World Health Organization. WHO Report on the Global Tobacco Epidemic, 2013: Enforcing Bans on Tobacco Advertising, Promotion and Sponsorship. Geneva: WHO; 2013. Available at http://www.who.int/tobacco/global_report/2013/en/. Accessed 18 Nov 2016.

[14] Khan JA, Amir Humza Sohail AM, Arif Maan MA (2016). Tobacco control laws in Pakistan and their implementation: a pilot study in Karachi. J Pak Med Assoc.

[15] Siddiqi K, Scammell K, Huque R, Khan A, Baral S, Ali S, Watt I (2016). Smokeless tobacco supply chain in south asia: a comparative analysis using the WHO framework convention on tobacco control. Nicotine Tob Res.

[16] Khan A, Huque R, Shah SK, Kaur J, Baral S, Gupta PC, Cherukupalli R, Sheikh A, Selvaraj S, Nargis N, Cameron I, Siddiqi K (2014). Smokeless tobacco control policies in south asia: a gap analysis and recommendations. Nicotine Tob Res.

[17] Nishtar S, Mirza Z, Mohamud KB, Latif E, Ahmed A, Jafarey NA (2004). Tobacco control: National Action Plan for NCD Prevention, Control and Health Promotion in Pakistan. J Pak Med Assoc.

[18] Chadda R, Sengupta S (2002). Tobacco use by Indian adolescents. Tob Induc Dis.

[19] Jason LA, Hunt YM, Adams ML, Pokorny SB, Gadiraju PB (2007). Strengthening communities’ youth access policies may facilitate clean indoor air action. Prev Chronic Dis.

[20] Rodu B, Godshall WT (2006). Tobacco harm reduction: an alternative cessation strategy for inveterate smokers. Harm Reduct J.

